# Popliteus impingement after TKA may occur with well-sized prostheses

**DOI:** 10.1007/s00167-016-4330-8

**Published:** 2016-09-26

**Authors:** Michel P. Bonnin, Arnoud de Kok, Matthias Verstraete, Tom Van Hoof, Catherine Van der Straten, Mo Saffarini, Jan Victor

**Affiliations:** 1 Centre Orthopédique Santy, 24 Av Paul Santy, Lyon, France; 2 Hopital Privé Jean Mermoz, 55 Av Jean Mermoz, 69008 Lyon, France; 3 Department of Orthopaedics, Ghent University, De Pintelaan, 185, Ghent, Belgium; 4 Accelerate Innovation Management, Rue de Hollande 4-6, 1204 Geneva, Switzerland

**Keywords:** Popliteus tendon, TKA pain, TKA sizing, TKA impingement

## Abstract

**Purpose:**

To determine the mechanisms and extents of popliteus impingements before and after TKA and to investigate the influence of implant sizing. The hypotheses were that (1) popliteus impingements after TKA may occur at both the tibia and the femur, and (2) even with an apparently well-sized prosthesis, popliteal tracking during knee flexion is modified compared to the preoperative situation.

**Methods:**

The location of the popliteus in three cadaver knees was measured using computed tomography, before and after implantation of plastic TKA replicas, by injecting the tendon with radiopaque liquid. The pre- and post-operative positions of the popliteus were compared from full extension to deep flexion using normosized, oversized, and undersized implants (one size increments).

**Results:**

At the tibia, TKA caused the popliteus to translate posteriorly, mostly in full extension: 4.1 ± 2 mm for normosized implants, and 15.8 ± 3 mm with oversized implants, but no translations were observed when using undersized implants. At the femur, TKA caused the popliteus to translate laterally at deeper flexion angles, peaking between 80° and 120°: 2 ± 0.4 mm for normosized implants and 2.6 ± 0.5 mm with oversized implants. Three-dimensional analysis revealed prosthetic overhang at the posterosuperior corner of normosized and oversized femoral components (respectively, up to 2.9 mm and 6.6 mm).

**Conclusions:**

A well-sized tibial component modifies popliteal tracking, while an undersized tibial component maintains more physiologic patterns. Oversizing shifts the popliteus considerably throughout the full arc of motion. This study suggests that both femoro- and tibio-popliteus impingements could play a role in residual pain and stiffness after TKA.

**Electronic supplementary material:**

The online version of this article (doi:10.1007/s00167-016-4330-8) contains supplementary material, which is available to authorized users.

## Introduction

Residual pain and poor outcomes after total knee arthroplasty (TKA) can be attributed to soft-tissue impingements, which could arise due to prosthetic overhang at the femur [10, 30] or the tibia [9]. Impingements may involve various anatomic structures such as the medial collateral ligament (MCL), the iliotibial band, the popliteus tendon, the patellar tendon, and the medial and lateral patellar retinaculum [3, 8, 10, 30]. The popliteus tendon is of special interest due to its intra-articular location and its close contact with the posterolateral tibial plateau and the lateral condylar margin [46].

**Fig. 1:**
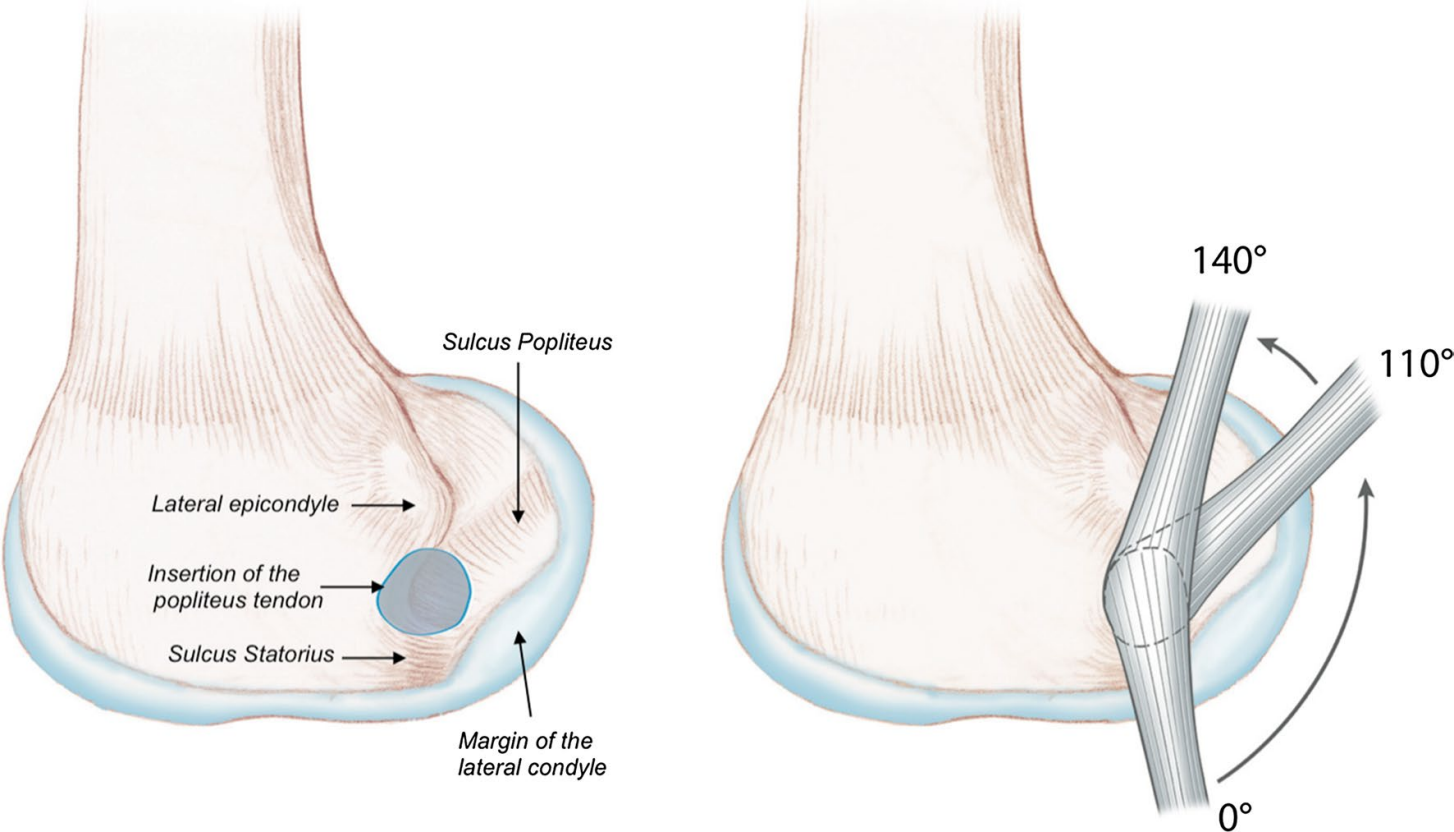
In native knees, the popliteus tendon inserts in an area located distal to the lateral epicondyle. In extension, the tendon is seated in the sulcus statorius. During flexion, it glides along the margin of the lateral condyle and then seats in the popliteus sulcus beyond 100° of flexion

In a normal knee, the popliteus remains in close contact with the convex posterolateral area of the lateral tibial plateau, up to the popliteus hiatus, where it is stabilized by the popliteomeniscal fascicles [11, 16, 38, 43, 46, 47]. It then crosses the margin of the lateral condyle and inserts anterior and distal to the lateral epicondyle [24, 29, 32, 44]. In full extension, the popliteus is engaged in a distal indentation of the lateral condyle, called the sulcus statorius [43]. During flexion, it glides over the bumpy margin of the lateral condyle, and beyond 100° of flexion, it lies entirely within the groove of the sulcus popliteus (Fig. 1) [28, 44].

**Fig. 2:**
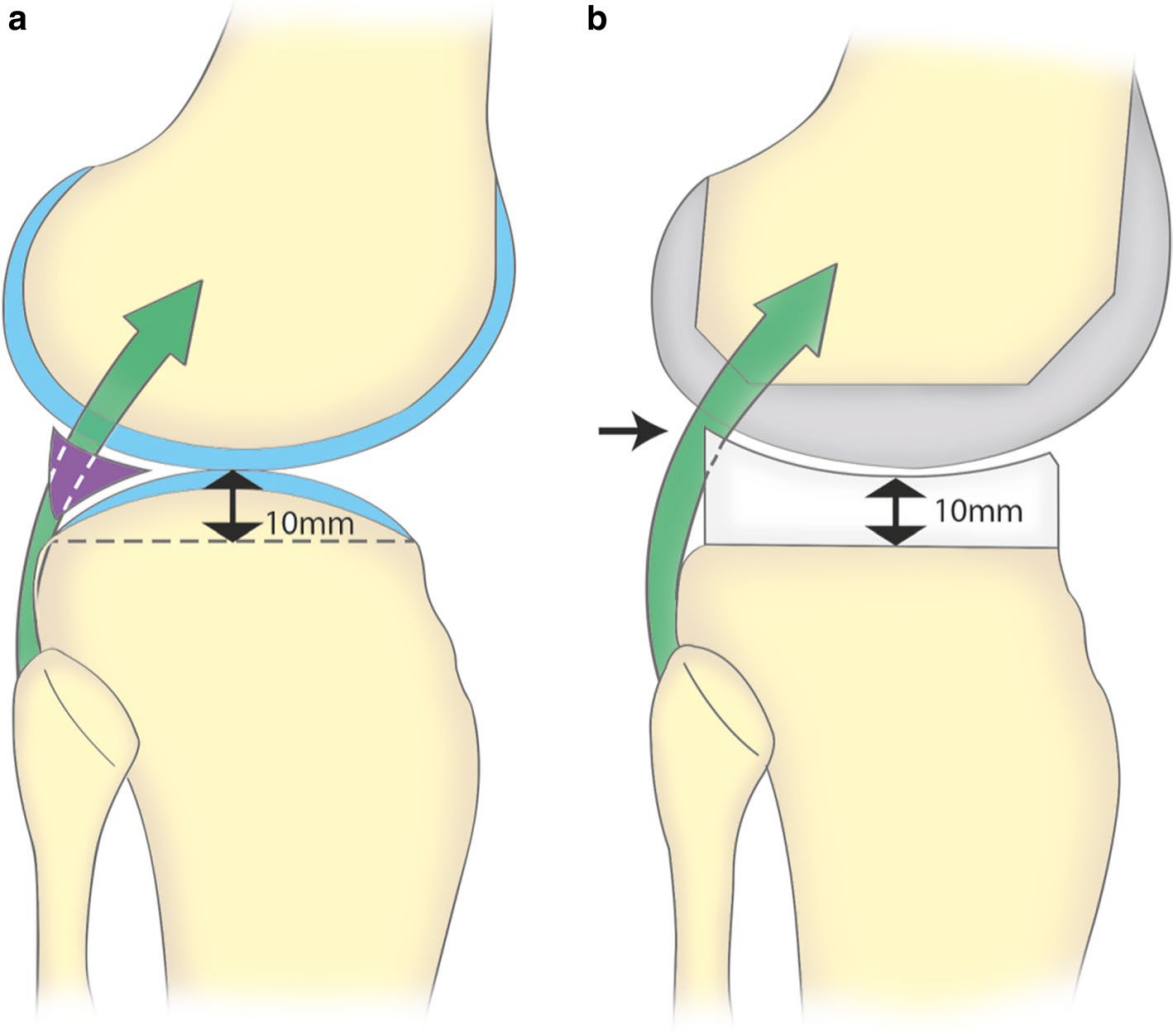
In a normal knee, (**a**) the lateral tibial plateau is convex in the sagittal plane. The popliteus (*green*) is in contact with the posterolateral margin of the plateau and passes through the popliteus hiatus within the meniscus (*purple*). The tibial resection in TKA (*dashed line*) is typically performed 10 mm below the convexity of the plateau. After TKA, (**b**) the PE *insert* does not reproduce the* convex shape* of the native plateau and impingement occurs at the posterosuperior border of the base plate

In a TKA, the thickness of the tibial component is selected to restore the joint line and to match the contours of the resected surfaces [13, 14, 31, 36]. Therefore, a superstructure of polyethylene is generally built above the posterolateral area of the tibial plateau, leading to a potential risk of popliteus impingement (Fig. 2). At the femur, any shape difference between the prosthetic and the native lateral condylar margin, such as induced by the design, the sizing or the positioning of the femoral component, potentially affects the tracking of the popliteus [4, 45]. Indeed, impingements have been reported secondary to friction against femoral osteophytes or overhanging prosthetic condyles [1, 4, 26] and have been successfully treated by arthroscopic popliteus release [1].

It has been demonstrated that mediolateral overhang of the femoral component could cause residual pain after TKA, and that slightly undersizing the femoral component may improve pain scores [10, 30]. However, undersizing may lead to implant subsidence or tibiofemoral instability [5] and could compromise bone–implant fit [7]. Many recent femoral components are available in ‘standard’ or ‘narrow’ versions and allow greater tibia–femur size mismatch that enables surgeons to fine-tune mediolateral sizing. Nevertheless, the optimal sizing, exact fit or slight under-coverage, remains controversial.

The purpose of this study was to determine the mechanisms and extents of popliteus impingements by examining the bony and prosthetic contours of knees before and after TKA and to investigate the influence of implant sizing. The study hypotheses were that (1) popliteus impingements after TKA may occur at both the tibia and the femur, and (2) even with an apparently well-sized prosthesis, the position or tracking of the popliteus during knee flexion is modified compared to the preoperative situation. A validation (or invalidation) of these hypotheses might have clinical consequences concerning the optimal sizing and positioning of the prosthetic components and the design evolution of the components towards a more ‘popliteus friendly’ design.

## Materials and methods

The location of the popliteus tendon was studied on three fresh frozen cadaver knees throughout the flexion–extension range, using computed tomography (CT), before and after implantation of TKA. The cadavers had been donated for research by testament, and none of the cadaver knees had history of previous surgery.

### Specimen preparation

With a lateral longitudinal approach, the iliotibial band was incised and the popliteus tendon was dissected from the condyle to the musculotendinous junction. A mixture of glycerol (60 %) and barium sulphate (40 %) was injected in the popliteus from its insertion to render the entire tendon radiopaque and enable its visualization in isolation from surrounding soft tissues. This technique was described for imaging of the posterior cruciate ligament [49].

The three knees were then scanned using a 64-slice multidetector CT scanner (Siemens Sensation, Munich, Germany) with the lower limb in supine position and included the femoral head and the ankle to calculate the mechanical tibiofemoral angle (TFA). The knees were then scanned using 0.6-mm-thick slices from full extension to full flexion in 20° increments.

### TKA implantation

The TKA implants used were plastic replicas of the fixed-bearing postero-stabilized HLS KneeTech^®^ (Tornier SA, Montbonnot, France). The specimens were produced by the manufacturer using rapid prototyping: Fused Deposition Modelling FDM^®^ with a Stratasys Dimension Elite™ machine (Eden Prairie, MN, USA) from a non-radiopaque and non-magnetic polymer (Acrylonitrile butadiene styrene). Implantation was performed through a medial parapatellar approach using the conventional instrumentation for a tibia first technique with orthogonal cuts and a posterior referencing technique for the resection of the posterior condyles. The femoral component was aligned with the surgical transepicondylar axis (TEA), and the tibial component was aligned with the centre of the anterior tibial tuberosity. Implants were cemented with barium-free polyester (Polyester Demaere, Brussels, Belgium).

Specimen #1 was implanted with a ‘normosized’ TKA, where the contour of the tibial component fits almost exactly with the tibial cortex and where the femoral components did not overhang the bony contours in any visible area of the bone cuts. Specimen #2 was implanted with an ‘undersized’ TKA (one size smaller), with the contour of the tibial base plate about 3 mm inside the tibial cortex and with a 3 mm border of non-covered resected bone at the posterior portion of the distal femoral cut. Specimen #3 was implanted with an ‘oversized’ TKA (one size larger), with the tibial implant overhanging the bony contour of the lateral tibial plateau by about 3 mm and the femoral component overhanging of about 3 mm at the anterodistal area (anterior chamfer).

### CT scan analysis

**Fig. 3:**
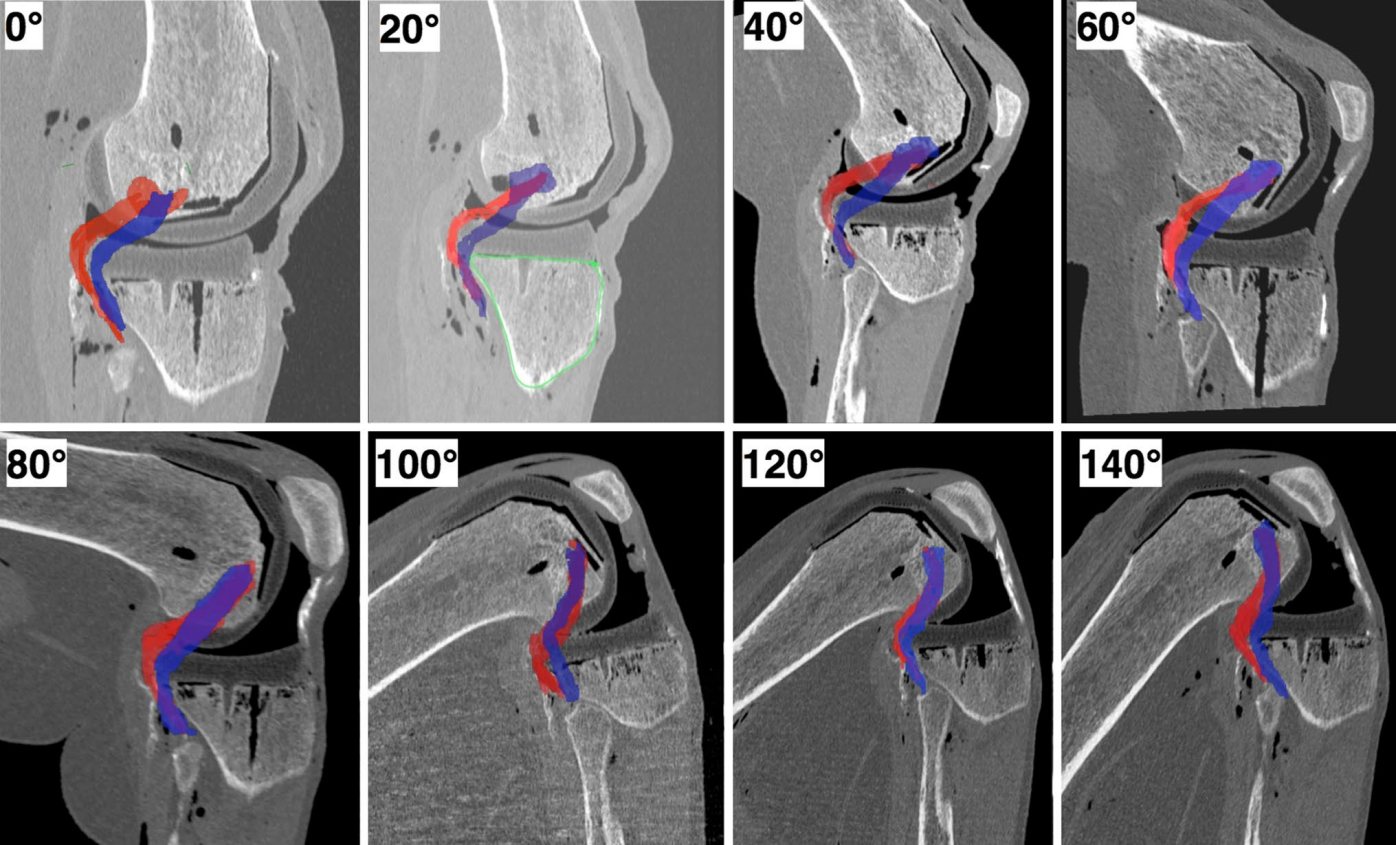
Imaging of the popliteus tendon from raw DICOM images, in a native (preoperative, *blue*) knee and in an implanted (post-operative, *red*) knee with an oversized component

**Fig. 4:**
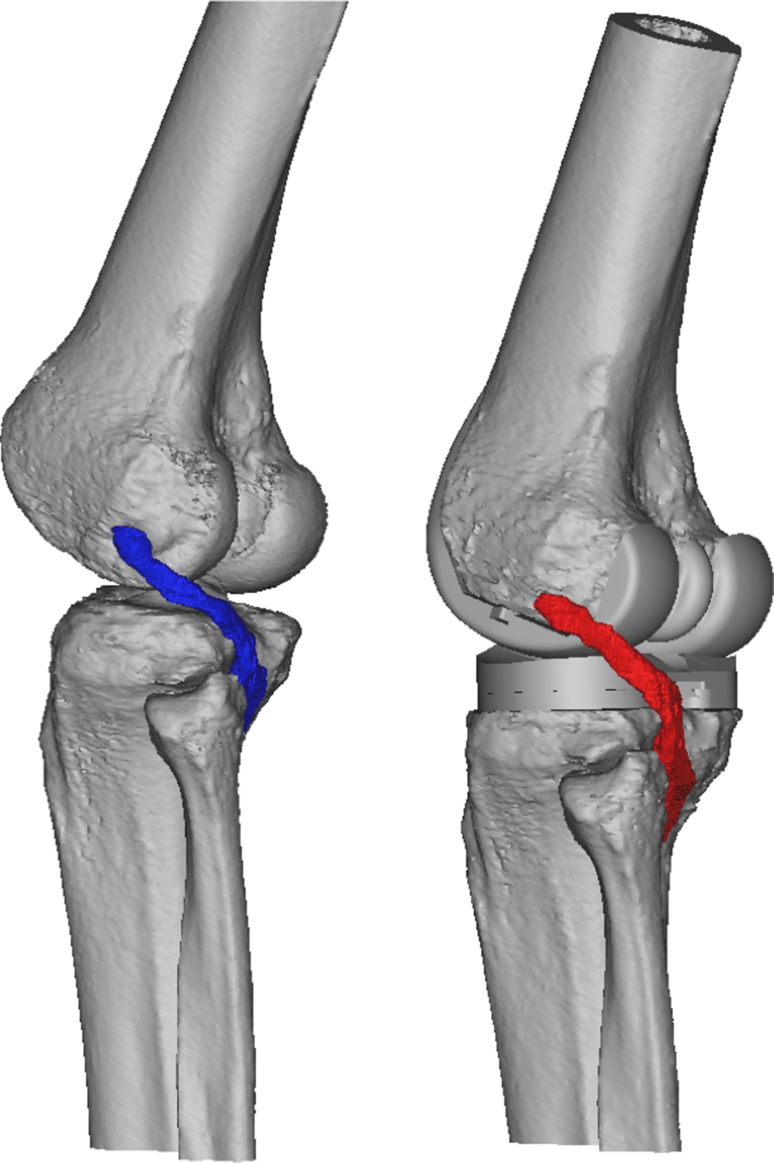
Three-dimensional reconstruction of the knee, before and after implantation of a ‘normosized’ TKA. The popliteus crosses the posterolateral aspect of the tibial plateau. Bone reconstructions were obtained using Mimics^®^ software (Materialize^®^), and implant models (STL files) were superposed

Post-operatively, the full lower limb was scanned following the same imaging protocol used for the preoperative scans, to verify that the final alignment was in the range 180° ± 3°. Raw DICOM images enabled visualization of the popliteus during flexion before and after TKA implantation (Fig. 3). From these raw DICOM images, the popliteus was digitized by manual segmentation at each slice level, using Mimics^®^ software (Materialize^®^, Leuven, Belgium) in order to generate three-dimensional (3D) reconstructions. Stereolithography files (STL) of the implants obtained from the manufacturer were superposed with the raw DICOM images (Fig. 4). Coordinates of digitized points were exported to spreadsheets and processed using MATLAB^®^ (MathWorks^®^, Natick, MA, USA).

### Tibial coordinate system

The tibial coordinate system was established with its origin at the centre of the tibial keel at the level of the tibial cut, which defined the transverse plane. The anteroposterior axis intersects the origin, perpendicular to the posterior tibial margin; the mediolateral axis was parallel to the posterior tibial margin intersecting the origin, and the proximodistal axis was perpendicular to the transverse plane intersecting the origin.

**Fig. 5:**
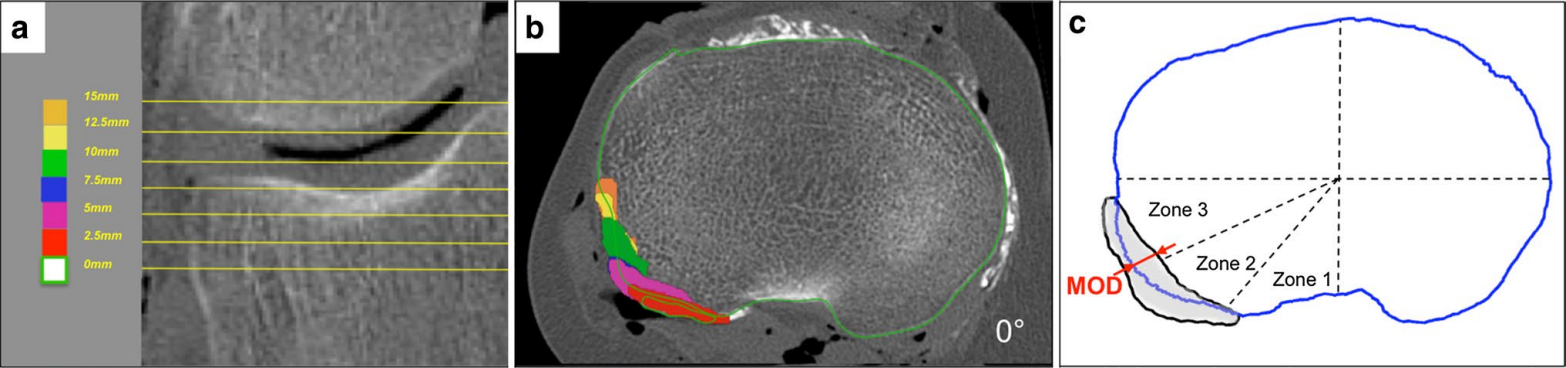
CT scan of the knee at 0° of flexion: **a** coronal view indicating different slices analysed (0 mm corresponds to the tibial resection level; 10 mm corresponds to the joint line); **b** transverse view at the level of tibial resection illustrating the position of the popliteus at different levels above; **c** representation of the entire transverse area covered by the popliteus (*grey*) obtained using Matlab^®^. The maximum overlap distance (MOD, red arrow) was measured separately in three sectors of the posterolateral quadrant: Zone 1 (0°–30°), Zone 2 (30°–60°), and Zone 3 (60°–90°)

The overlap of the popliteus on the native tibial plateau was measured at the level of the tibial cut after superimposing the popliteus, as seen on each CT slice. The ‘maximum overlap distance’ (MOD) was measured between the cortical contour of the plateau and the inner point of the popliteus in three distinct zones (Fig. 5).

**Fig. 6:**
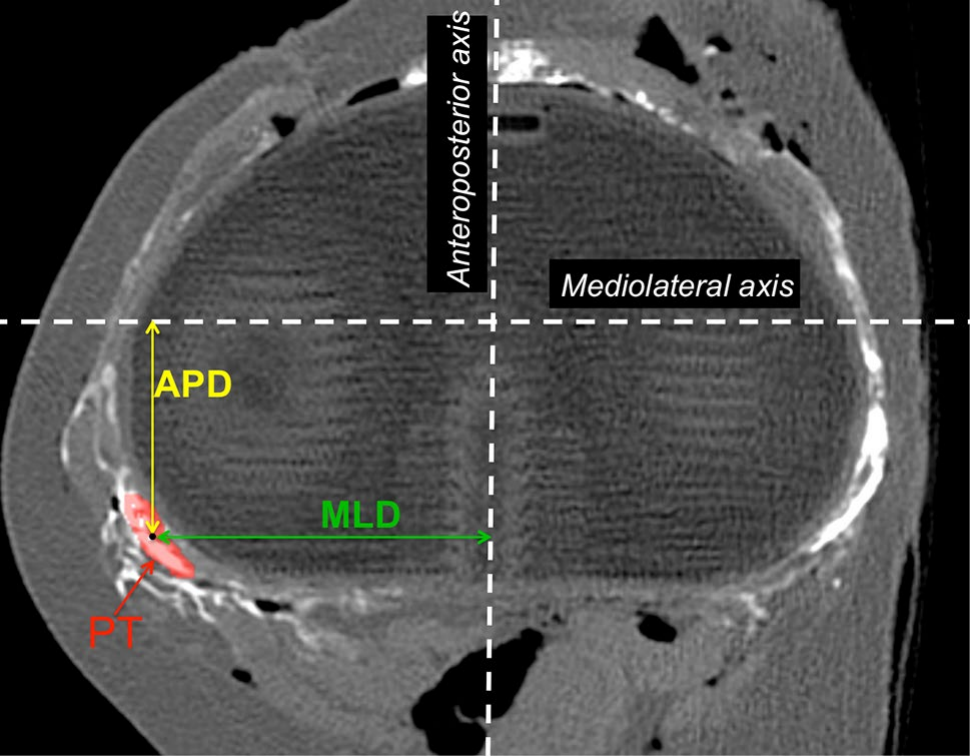
Geometric centre of the popliteus was used to determine the anteroposterior distance (APD) and mediolateral distance (MLD) with respect to the origin. The anteroposterior distance (APD) and mediolateral distance (MLD) were measured in the transverse plane from the origin of the tibial coordinate system orthogonally to the geometric centre of the popliteus (*red*)

The pre- and post-operative positions of the popliteus were analysed and compared on each slice in the transverse plane, from full extension (0°) to full flexion (140°) (Fig. 6). The pre- and post-operative translations of the popliteus were measured in the entire area of the prosthetic plateau (cf. additional material), with a special focus at 0 mm (tibial cut), 5 mm (middle of the prosthetic tibial plateau), and 10 mm (superior border of the plateau).

### Femoral coordinate system

**Fig. 7:**
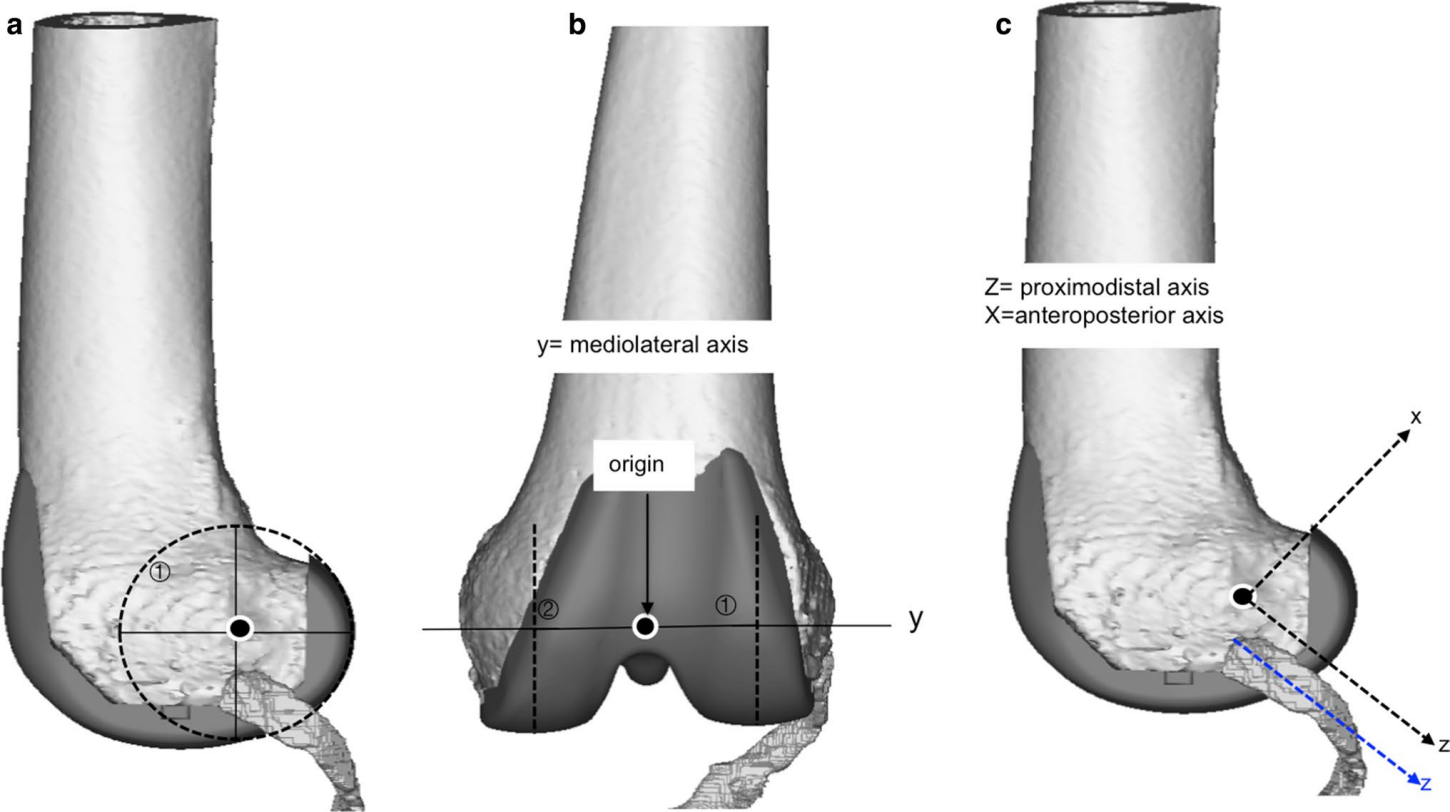
Femoral coordinate system was established with the mediolateral axis as the line that intersects the centres of the *circles* that best fit the femoral condyles (**a**). The origin was defined as the mid-point between the medial and lateral femoral cortices along the mediolateral axis (**b**). The proximodistal axis was set parallel to the popliteus tendon, between its femoral insertion and the point where it crosses the lateral condylar margin, and the anteroposterior axis was perpendicular to the popliteus tendon at its femoral insertion (**c**)

The femoral coordinate system (Fig. 7) was established with the mediolateral axis being the line that intersects the centres of the circles that best fit the femoral condyles. The origin was defined as the mid-point between the medial and lateral femoral cortices along the mediolateral axis. The proximodistal axis was set parallel to the popliteus tendon, between its femoral insertion and the point where it crosses the lateral condylar margin. The frontal and transverse planes, perpendicular, respectively, to the anteroposterior and proximodistal axes, remained unchanged relative to the popliteus during knee flexion (static) but moved relative to the femur during flexion (dynamic). Only the sagittal plane remained unchanged relative to both the femur and the popliteus throughout flexion.

**Fig. 8:**
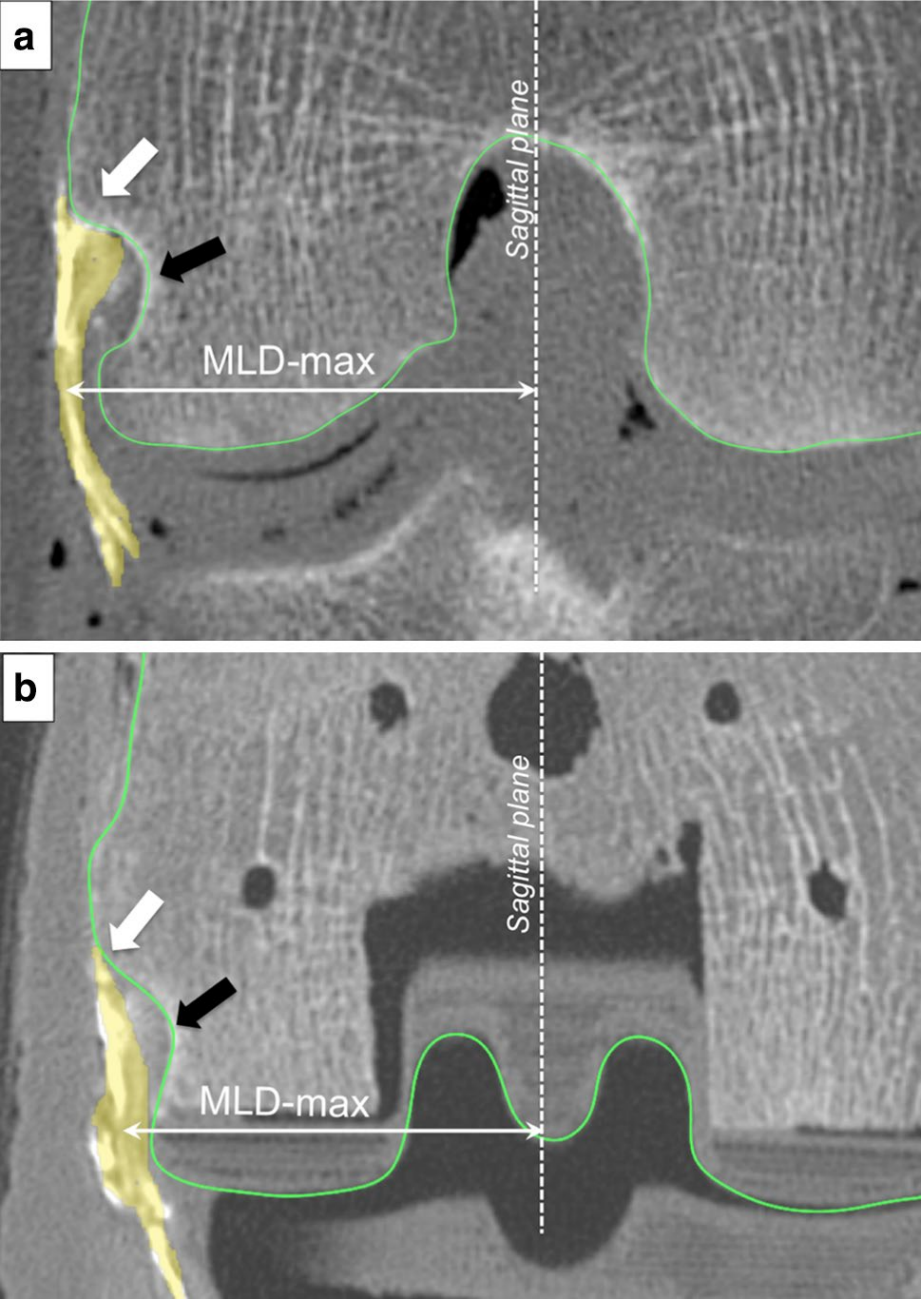
Maximum mediolateral distance (MLD-max) was measured at the apex of the lateral condylar margin, where the risk of prosthetic impingement is greatest. Measurements before (**a**) and after (**b**) TKA implantation. The popliteus is coloured in *yellow*, and the bony or prosthetic contours are outlined in *green*. The *white* arrow indicates the femoral insertion of the popliteus, and the black arrow points to the popliteus sulcus. The maximum mediolateral distance (MLD-max) was measured at the apex of the lateral condylar margin

The pre- and post-operative mediolateral positions of the popliteus were measured in the transverse plane, as distances from the sagittal plane to the geometric centre of the popliteus. Measurements were repeated at all flexion angles, from the femoral insertion of the popliteus to the joint line, with a special interest in the area where the popliteus crosses the condylar margin (Fig. 8).

Our institutional review board granted ethical approval for this study (Reference number EC-2014/0847, Ghent University, Ghent, Belgium).

### Statistical analysis

Inter- and intra-observer repeatability was determined using 40 measurements performed by three different observers. For inter- and intra-observer testing, the interclass correlation coefficients were, respectively, *r* = 0.82 and *r* = 0.83. Statistical analyses were conducted using SPSS software (IBM, Armonk, NY, USA).

## Results

### Tibia–popliteus relationships

**Fig. 9:**
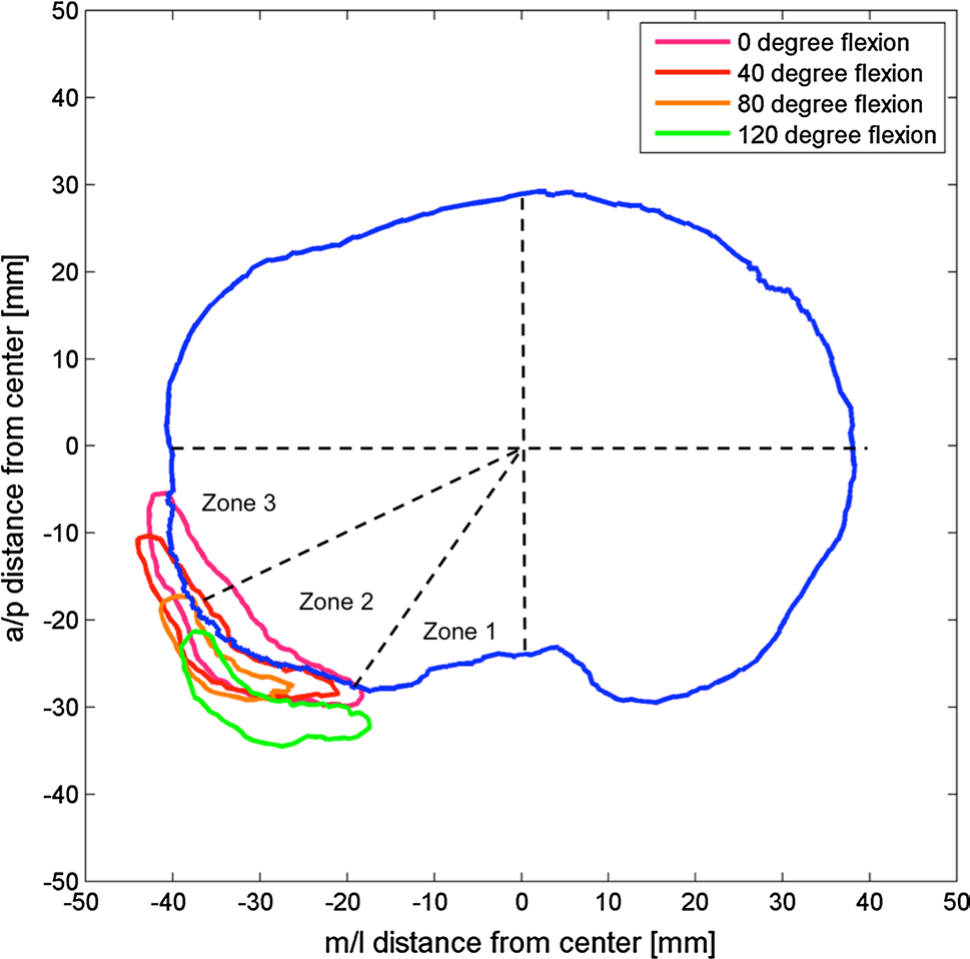
Projection of the popliteus on the tibial plateau at the resection level throughout the range of flexion in the native knee. In extension (*pink*), the tendon overlaps the contour of the tibial plateau considerably, whereas at 40° flexion (*red*), the overlap is minimal. At 80° flexion (*orange*) and 120° flexion (*green*), the popliteus never overlaps the tibial plateau

In native knees (Fig. 9; Table 1), the popliteus overlapped the posterolateral aspect of the tibial plateau in Zones 2 and 3, between full extension and 40° of flexion. No overlap was observed in Zone 1 throughout the flexion range. The maximum overlap distance (MOD) was 4.8 mm observed in Zone 3, but some inter-specimen variability was observed.

**Table 1:** Maximum overlap distance (mm)

Flexion angle	Zone 2			Zone 3		
	Mean ± SD	Max	Median	Mean ± SD	Max	Median
0°	1.2 ± 1.3	4.1	0.3	1.4 ± 2.3	4.8	0.4
20°	1.3 ± 1.1	2.4	1.3	3.1 ± 1.0	2.5	0.1
40°	0.5 ± 0.5	1.0	0.5	0.4 ± 0.6	1.3	0.2
60°	0.2 ± 0.4	0.9	0.0	0.0 ± 0.6	0.1	0.0
80°	0.0 ± 0.0	0.0	0.0	0.1 ± 0.1	0.2	0.1
100°	0.1 ± 0.3	0.5	0.0	0.0 ± 0.0	0.0	0.0
120°	0.3 ± 0.5	1.0	0.0	0.0 ± 0.0	0.0	0.0
140°	0.0 ± 0.0	0.0	0.0	0.0 ± 0.0	0.0	0.0

**Fig. 10:**
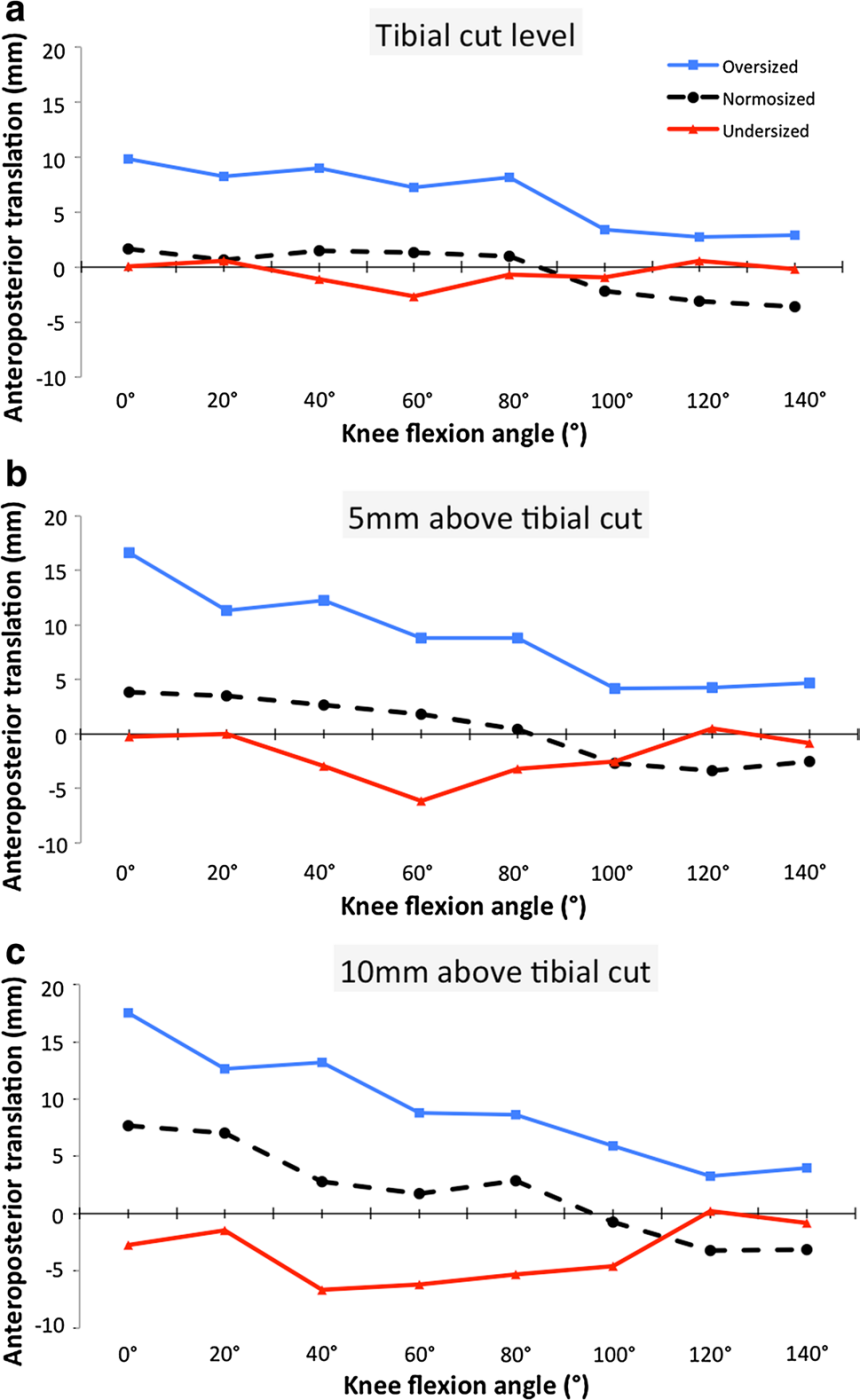
Posterior translation of the popliteus after TKA compared to its native position (*vertical* axis) throughout the range of knee flexion at: **a** the tibial cut level, **b** 5 mm above the tibial cut, and **c** 10 mm above the tibial cut

**Fig. 11:**
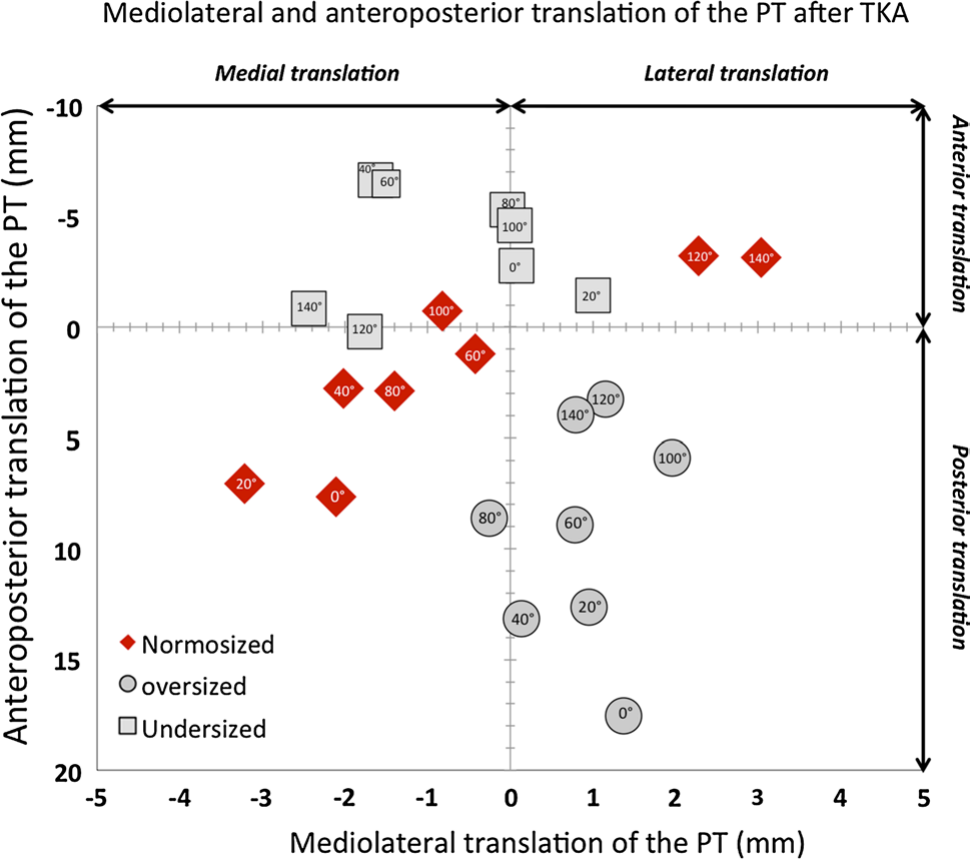
Graphic representation of anteroposterior and mediolateral translations of the popliteus at different flexion angles and for different implant sizes. In oversized TKA, the popliteus is displaced posteriorly and laterally, whereas in undersized TKA it is displaced medially and anteriorly

After implantation of a normosized TKA (Figs. 10, 11), the popliteus was posteriorly translated, from full extension to 100° of flexion, but an anterior translation was observed thereafter in deep flexion. The greatest deviations were observed at the superior tibial border, 10 mm proximal to the tibial cut. The mean posterior translation of the popliteus at the plateau level was 4.1 ± 2 mm (range 1.7–7.7) in full extension, and 3.5 ± 2.2 mm (range 0.7–7) at 20° of knee flexion. A medial translation of the popliteus was also observed between 0° and 100° of flexion, with a maximum of 3.1 mm at 20° of flexion and a lateral translation was observed in deep flexion with a maximum of 3 mm at 140° flexion. When an oversized TKA was implanted, a greater posterior translation was observed throughout the range of movement, at all levels of the prosthetic tibial plateau. The mean posterior translation of the popliteus was 15.8 ± 3 mm (range 9.8–19.5) in full extension and 4.3 ± 0.8 mm (range 2.9–5.3) in deep flexion. The tendon appeared also to be laterally translated throughout the range of flexion. When an undersized TKA was implanted, the popliteus was anteriorly translated during the entire range of flexion and a medial translation less than 2.5 mm was also observed.

### Femur–popliteus relationships

In native knees, the mediolateral position of the popliteus followed the bony contour of the lateral condyle. Between 0° and 40° of flexion, the popliteus disengaged from the sulcus statorius and translated slightly laterally and then medially by 3.5 ± 0.4 mm (range 2.9–3.9) between 40° and 120° of flexion, until it was fully seated into the popliteus sulcus.

**Fig. 12:**
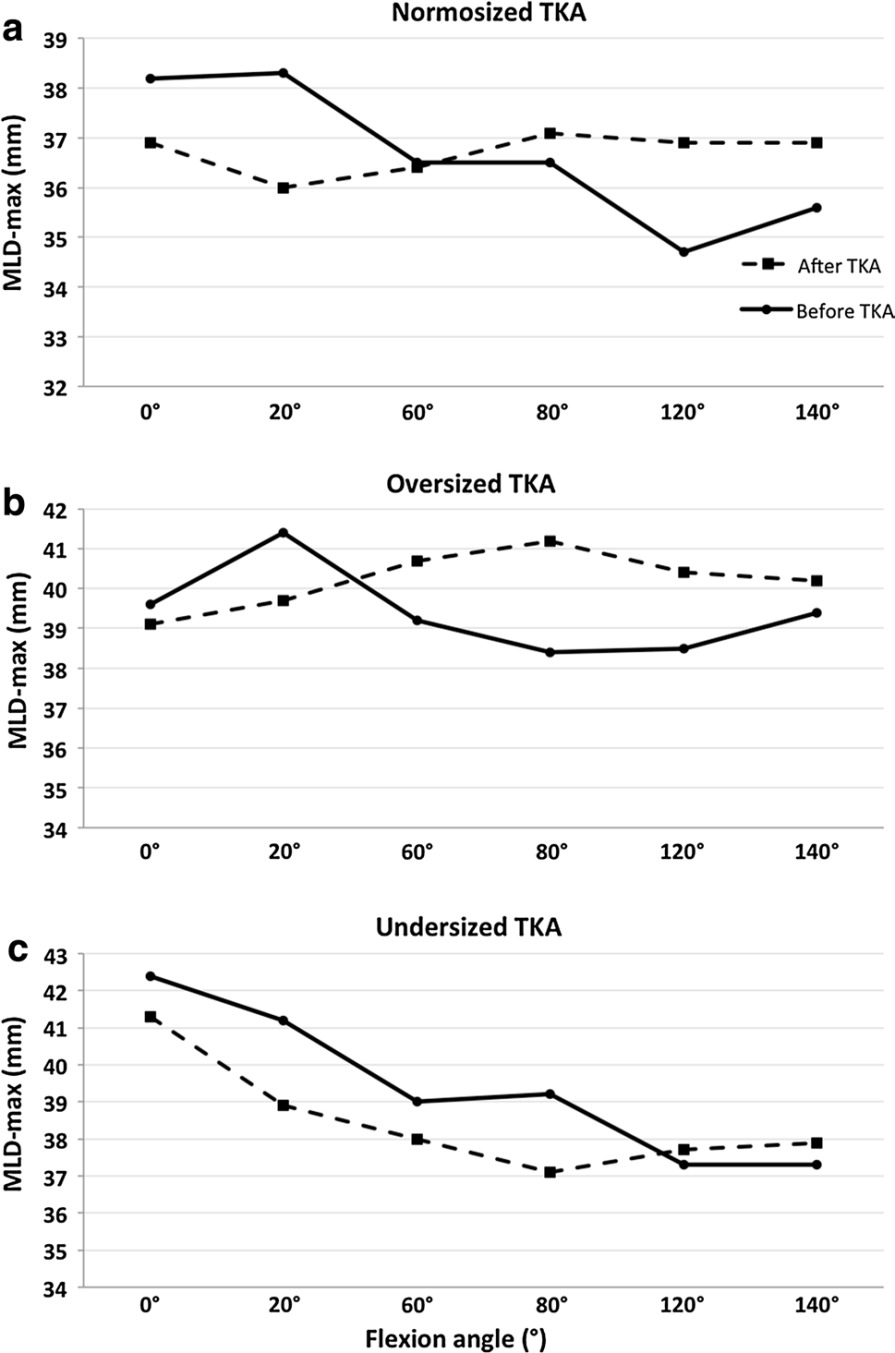
Maximum distance (MLD-max) between the sagittal plane and the geometric centre of the popliteus before (solid) and after (dashed) TKA implantation with normosized (**a**), oversized (**b**), and undersized (**c**) prosthesis

With a normosized TKA (Fig. 12), compared to the preoperative situation, the popliteus was more medial between full extension and 60° of flexion, beyond which it was lateralized until deep flexion. With an oversized TKA, the same pattern was observed with further lateralization compared to the normosized TKA. The maximum lateralization was observed at 80° of flexion, which then decreased progressively at greater flexion angles. With an undersized TKA, the popliteus was medialized from full extension to 120° of flexion compared to the preoperative knee. Three-dimensional analysis revealed prosthetic overhang at the posterosuperior corner of normosized and oversized femoral components (respectively, up to 2.9 mm and 6.6 mm), which could explain the ‘paradoxical lateralization’ of the popliteus during flexion (cf. Figure A2 in additional material).

## Discussion

The main finding of this study is that a well-sized tibial component modifies popliteal tracking, while an undersized tibial component maintains more physiologic patterns. The data also demonstrate that oversizing the tibial component by one size increment shifts the popliteus considerably throughout the full arc of motion. These results confirm previous clinical investigations that reported better pain scores in patients with ‘undersized’ implants [10], and poorer outcomes in patients with posterior tibial overhang [9]. Why an ‘anterior translation’ of the popliteus was observed in deep flexion with a normosized prosthesis remains unclear. An explanation could be that sacrificing the popliteo–meniscal fascicles during implantation, secondary to lateral meniscectomy, destabilizes the tendon in deep flexion [38, 47].

Another finding was that the popliteus–condyle contact is modified after TKA throughout the flexion range, whatever the sizing option, which resulted in a ‘reversed pattern’. From full extension to mid-flexion, the popliteus was medialized because the margin of the prosthetic lateral condyle does not reproduce the smooth ridge of the native lateral condyle, which is often removed during surgery. From mid to deep flexion, the tendon was lateralized due to prosthetic overhang at the lateral condyle.

Residual pain is a very frustrating situation after TKA, and identifying the cause can be challenging [40]. Soft-tissue impingements have been described as an aetiology but mostly in patients with overhanging components [9, 10, 30], and little is known about the behaviour of the soft tissues surrounding a well-sized TKA. There is little published literature on the diagnosis and treatment of popliteus impingement after TKA. While some authors reported successful pain relief after tendon release [1, 4, 26], none investigated the pathophysiology in detail. Allardyce et al. [1] reported results of arthroscopic release in two patients presenting ‘popliteus tendon dysfunction’ but did not describe the nature and location of the impingement. Likewise, Kazakin et al. [26] observed snapping popliteus tendon during TKA. The consequences of such popliteus tendon release in prosthetic knees are unclear. While De Simone et al. [15] reported lower function score, neither Kesman et al. [27] nor Ghosh et al. [20] observed adverse effects after popliteus transection, in vivo or in vitro. Recently, Cottino et al. [12] reported an increased TKA laxity after popliteus section, both with cruciate-retaining and postero-stabilized prostheses.

To avoid popliteus impingements after TKA, slightly undersizing the tibial component could be an option, in order to preserve a peripheral bony margin at its posterolateral corner. It must be noted, however, that excessive undersizing could lead to implant subsidence and failure [5]. The use of anatomic base plates, which replicate the tibial asymmetry, may help the surgeon to both undersize laterally and preserve a good medial coverage. Dai et al. [13] and Martin et al. [31] recently demonstrated that asymmetric tibial base plates provide better conformity to resected surfaces. Theoretically an ideal TKA should closely reproduce the shape of the resected articular surfaces, but this is difficult to achieve at the tibia because the polyethylene must respect a degree of congruency with the prosthetic condyles [18, 21, 25] and the lateral compartment presents high geometric variability [8, 18, 37, 41]. Consequently, none of the current TKA designs reproduce the convex shape of the posterolateral tibial plateau, though some lateral UKA models feature ‘dome-shaped’ tibial base plates with bi-concave polyethylene inserts, to better reproduce natural anatomy and kinematics [2, 48].

This study reports that a posterolateral condyle overhang may occur even with normosized implants. The reality of this phenomenon may be criticized as it has been infrequently reported in the literature. Hirakawa et al. [22] in a series of 40 TKAs in a Japanese population reported a overhang of the posterolateral condyle greater than 3 mm in 25 patients and suggested to reduce the dimensions of the posterolateral condyle in TKA. Shah et al. [42] in an Indian population also reported overhang of the posterolateral condyle when implanting a standard TKA. Mahoney et al. [30] measured intra-operatively overhang with the Scorpio prosthesis in several zones of the femur but did not detail the incidence of posterior condyle overhang. However, it must be noted that in Mahoney’s series, patients were operated via subvastus approach, which limits visualization in the posterolateral area.

Prosthetic posterior condyles implanted in this experiment were symmetric, as most TKAs available on the market. The shape of the posterior condyles has been investigated mostly in the sagittal plane [23, 33, 39, 51], and little is known about their morphometry (symmetric or asymmetric) and their mediolateral dimensions. Recently, Monk et al. [35] analysed a series of MRI in healthy volunteers and demonstrated that the posterolateral condyle (mean width, 24 ± 3.5 mm) was narrower than the posteromedial condyle (mean width, 26 ± 3.0 mm), but further morphometric investigations are required to improve our understanding of this specific topic.

This study had some limitations: First, even if this study was based on pre- and post-operative comparison, the limited sample size remains a limitation of this study and anatomic variations may modify the bone–popliteus or implant–popliteus relationships that we observed. Second, only one implant design was used, and it is unclear whether our conclusions can be extended to other implants. It would be valuable to do this investigation with other designs such as medial-pivot or asymmetric TKAs. The posterior translation of the tendon may also be influenced by prosthetic kinematics, and it could be useful to compare postero-stabilized, cruciate-retaining and deep-dished TKAs [34, 50]. Third, this was a non-weight-bearing investigation, which could limit our conclusions, though by virtue of conformity of articular surfaces in postero-stabilized TKA, it can be assumed that the contact of the popliteus with bone and implant surfaces is similar in weight-bearing [6, 52, 53]. Fourth, it is a cadaveric work, and even with fresh specimens, one may argue that the behaviour of the soft tissues was altered. Fifth, the use of CT scans required injecting the popliteus via a lateral incision, which can modify its elasticity. In a preliminary investigation, the authors tested MRI, which proved inaccurate due to technical problems such as mirror images in deep flexion, poor-quality images due to temperature variations within specimens, small diameter of the MRI tube, which does not accommodate the knee in flexion. Finally, the study did not analyse the strain in the popliteus because inserting strain gauges [19] would compromise the image quality by scattering and even the use of chromium steel spheres precludes good soft-tissue analysis [17].

## Conclusion

This work demonstrates that sizing in TKA is challenging due to the non-anatomic design of current implants. It suggests that tibio-popliteus impingement might play a role in residual pain after TKA. The practical teaching is that surgeons should choose the size of the tibial implant considering not only the cortical contours of the resected tibia but also the volume of the implant. Hence, tibial components should be sized and positioned in a way to undercover by few mm the cortical contours of the lateral plateau. Design of tibial plateau should also be revisited in the future in order to avoid tibio-popliteus impingements.

## Electronic supplementary material

Below is the link to the electronic supplementary material.

The mean deviation of the popliteus tendon was measured on all CT slices covering the polyethylene tibial insert (TIFF 2372 kb)

The tibial coordinate system (TIFF 2616 kb)

The femur coordinate system (TIFF 3223 kb)

Three-dimensional analysis reveals prosthetic overhang, at the superolateral corner of the posterior condyle in a normosized femoral component, although the implant perfectly fits the bony contour at the distal cut level (TIFF 3134 kb)

Anteroposterior translations of the popliteus measured at the level of the polyethylene tibial insert (PDF 37 kb)

Mediolateral translations of the popliteus measured at the level of the polyethylene tibial insert (PDF 35 kb)

Dissection of the popliteus tendon and its movements during knee flexion in a normal knee (MOV 890954 kb)
