# Supplementary figures and images for: Popliteus impingement after TKA may occur with well-sized prostheses
Source: Knee Surg Sports Traumatol Arthrosc. 2016 Sep 26;25(6):1720–30. doi: 10.1007/s00167-016-4330-8 (PMC5487584; doi:10.1007/s00167-016-4330-8)

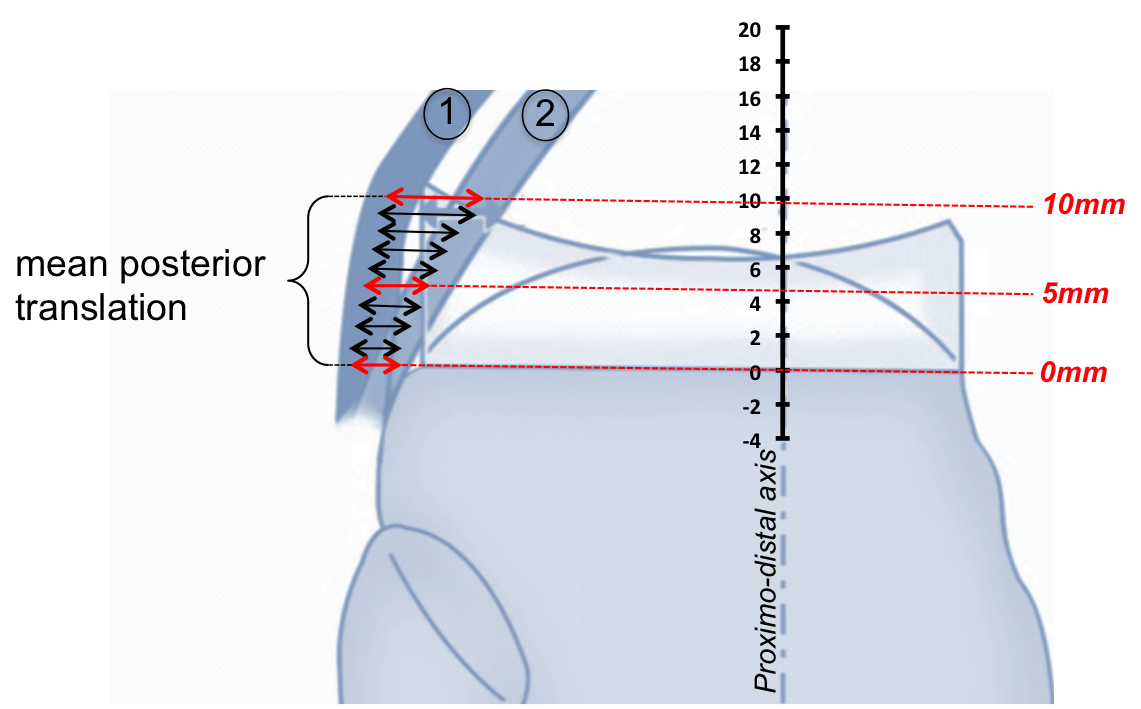

Supplement: Supplementary file 1 — The mean deviation of the popliteus tendon was measured on all CT slices covering the polyethylene tibial insert (TIFF 2372 kb) [file 167_2016_4330_MOESM1_ESM.tiff]

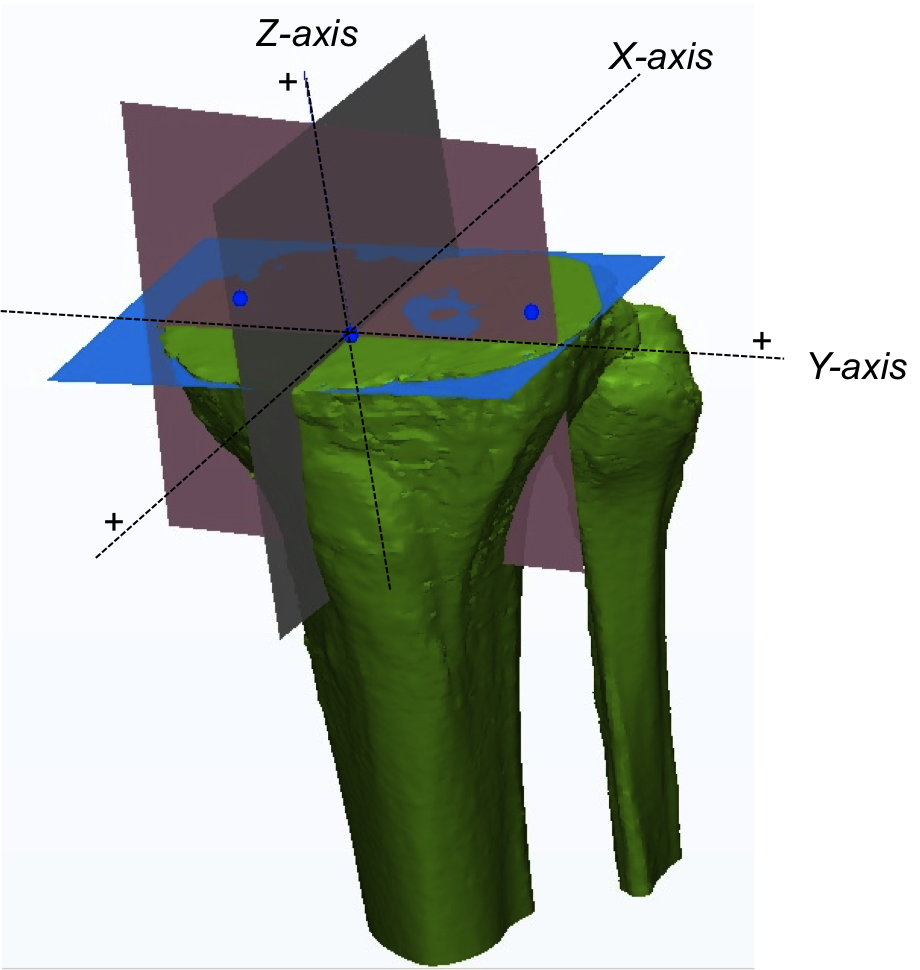

Supplement: Supplementary file 2 — The tibial coordinate system (TIFF 2616 kb) [file 167_2016_4330_MOESM2_ESM.tiff]

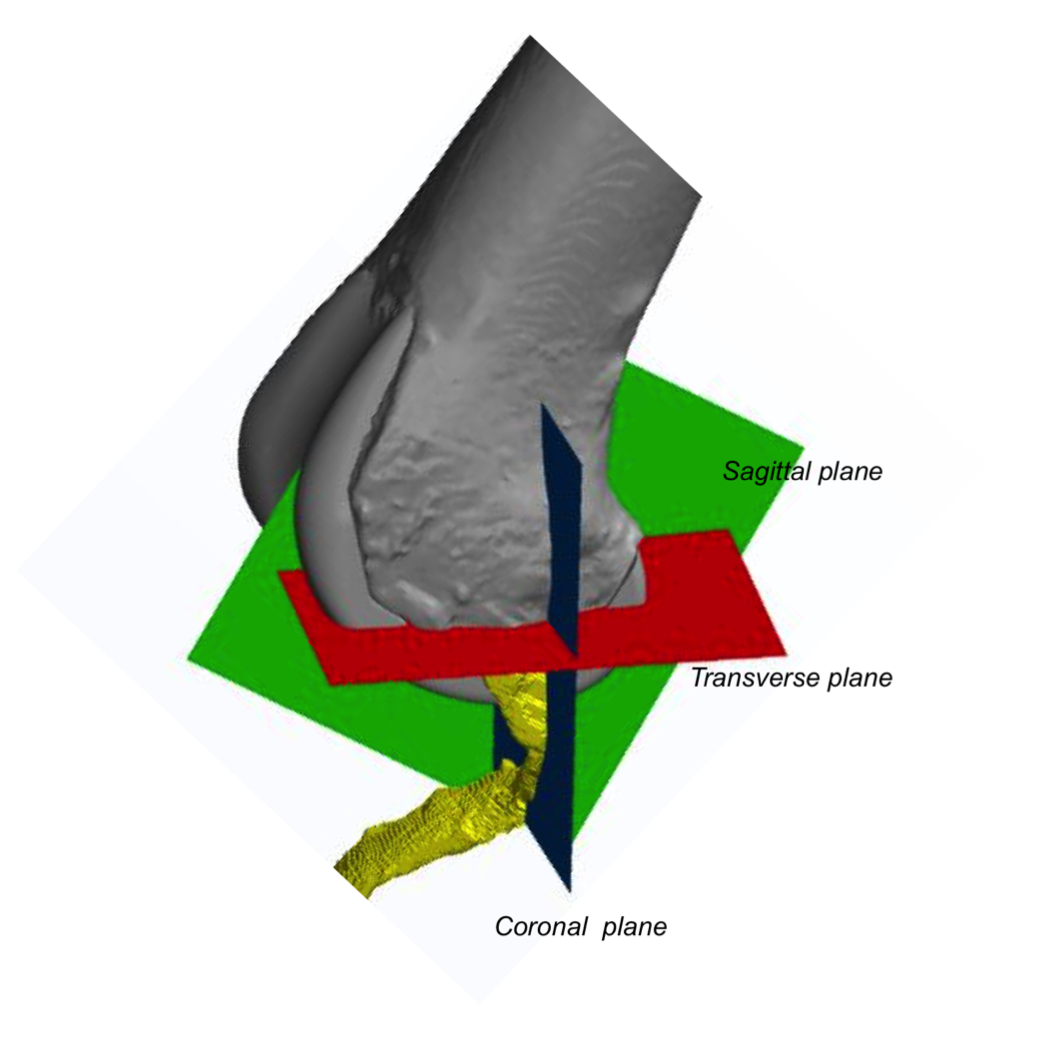

Supplement: Supplementary file 3 — The femur coordinate system (TIFF 3223 kb) [file 167_2016_4330_MOESM3_ESM.tiff]

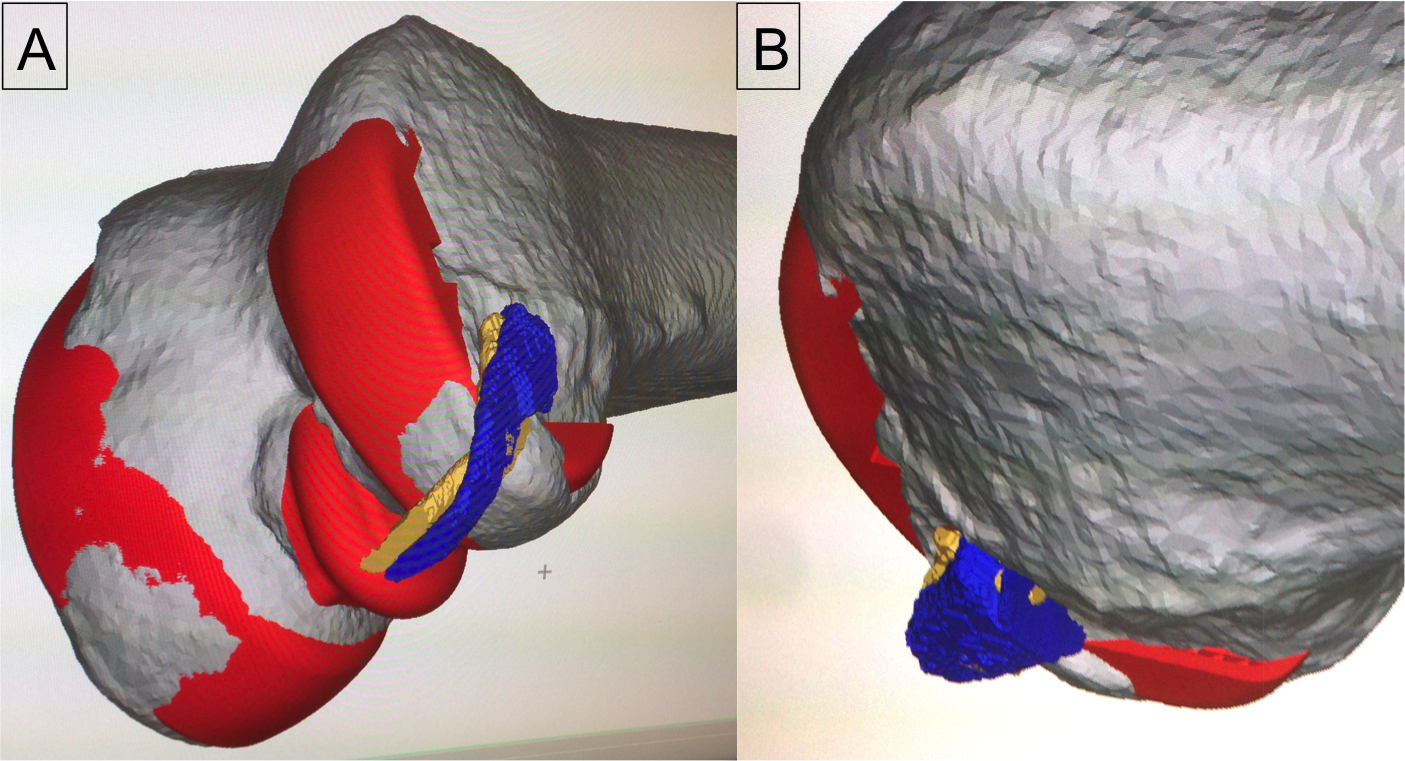

Supplement: Supplementary file 4 — Three-dimensional analysis reveals prosthetic overhang, at the superolateral corner of the posterior condyle in a normosized femoral component, although the implant perfectly fits the bony contour at the distal cut level (TIFF 3134 kb) [file 167_2016_4330_MOESM4_ESM.tiff]
