# Supplementary material for: Popliteus impingement after TKA may occur with well-sized prostheses
Source: Knee Surg Sports Traumatol Arthrosc. 2016 Sep 26;25(6):1720–30. doi: 10.1007/s00167-016-4330-8 (PMC5487584; doi:10.1007/s00167-016-4330-8)
Supplement: Supplementary file 5 — Anteroposterior translations of the popliteus measured at the level of the polyethylene tibial insert (PDF 37 kb) [file 167_2016_4330_MOESM5_ESM.pdf]

Table A1: Mean posterior translation of the popliteus tendon at the tibial plateau level (in mm*)

Flexion angle	Normosized TKA (1) mean ± SD	Normosized TKA (1) (Min – Max)	Oversized TKA (2) mean ± SD	Oversized TKA (2) (Min – Max)	Undersized TKA (3) mean ± SD	Undersized TKA (3) (Min – Max)
0°	4.1 ± 2.0	(1.7 – 7.7)	15.8 ± 3.0	(9.8 – 19.5)	-1.1 ± 1.2	(-3.0 – 0.1)
20°	3.5 ± 2.2	(0.7 – 7.0)	10.5 ± 2.0	(7.8 – 12.8)	-0.5 ± 1.0	(-1.8 – 0.7)
40°	2.7 ± 0.6	(1.5 – 3.3)	11.8 ± 1.3	(9.0 – 13.4)	-3.0 ± 1.8	(-6.6 – -1.7)
60°	1.7 ± 0.3	(1.2 – 2.0)	8.5 ± 0.7	(7.2 – 8.3)	-5.4 ± 1.3	(-6.5 – -2.6)
80°	1.2 ± 0.9	(0.5 – 2.9)	8.8 ± 0.3	(8.2 – 9.2)	-3.0 ± 1.6	(-5.3 – -0.6)
100°	-2.0 ± 0.7	(-2.7 – -0.7)	4.6 ± 0.9	(3.1 – 6.0)	-2.7 ± 1.4	(-4.6 – -0.9)
120°	-3.2 ± 0.4	(-3.7 – -2.6)	3.4 ± 0.7	(2.3 – 4.2)	0.4 ± 0.1	(0.2 – 0.6)
140°	-2.9 ± 0.5	(-3.6 – -2.1)	4.3 ± 0.8	(2.9 – 5.3)	-0.6 ± 0.4	(-1.0 – 0.0)

* positive value means posterior deviation
