# Supplementary material for: Popliteus impingement after TKA may occur with well-sized prostheses
Source: Knee Surg Sports Traumatol Arthrosc. 2016 Sep 26;25(6):1720–30. doi: 10.1007/s00167-016-4330-8 (PMC5487584; doi:10.1007/s00167-016-4330-8)
Supplement: Supplementary file 6 — Mediolateral translations of the popliteus measured at the level of the polyethylene tibial insert (PDF 35 kb) [file 167_2016_4330_MOESM6_ESM.pdf]

Table A2: Mean lateral translation of the popliteus tendon after TKA

Flexion angle	Normosized TKA mean ± SD	(Min – Max)	Oversized TKA mean ± SD	(Min – Max)	Undersized TKA mean ± SD	(Min – Max)
0˚	-3.8 ± 1.1	(-5.6 – -2.1)	-3.7 ± 2.6	(-7.6 – 1.4)	-2.5 ± 1.7	(-4.5 – 0.0)
20˚	-3.4 ± 0.9	(-4.6 – -2.0)	0.2 ± 0.5	(-0.5 – 0.9)	-0.2 ± 0.9	(-1.4 – 1.0)
40˚	-3.5 ± 0.9	(-4.9 – -2.0)	-2.0 ± 1.4	(-3.4 – 0.1)	-0.5 ± 1.3	(-1.6 – 1.9)
60˚	0.0 ± 0.7	(-0.9 – 1.9)	-1.0 ± 3.1	(-6.1 – 1.9)	0.4 ± 1.3	(-2.0 – 2.5)
80˚	-0.5 ± 0.7	(-1.4 – 0.6)	-2.7 ± 2.8	(-7.0 – -0.2)	0.8 ± 0.5	(0.0 – 2.5)
100˚	-0.9 ± 0.7	(-1.6 – 0.0)	0.5 ± 1.9	(-3.0 – 2.3)	-0.6 ± 0.9	(-1.7 – 0.8)
120˚	-0.5 ± 1.5	(-2.1 – 2.3)	-1.2 ± 2.5	(-5.2 – 1.7)	-1.3 ± 0.4	(-1.8 – -0.6)
140˚	-1.4 ± 2.4	(-3.7 – 3.0)	-0.9 ± 1.4	(-2.9 – 0.8)	-1.3 ± 0.5	(-2.4 – -0.8)
